# Computerized prediction of intensive care unit discharge after cardiac surgery: development and validation of a Gaussian processes model

**DOI:** 10.1186/1472-6947-11-64

**Published:** 2011-10-25

**Authors:** Geert Meyfroidt, Fabian Güiza, Dominiek Cottem, Wilfried De Becker, Kristien Van Loon, Jean-Marie Aerts, Daniël Berckmans, Jan Ramon, Maurice Bruynooghe, Greet Van den Berghe

**Affiliations:** 1 Department of Intensive Care Medicine, Katholieke Universiteit Leuven; Herestraat 49, B-3000 Leuven, Belgium; 2 Declarative Languages and Artificial Intelligence (DTAI), Department of Computer Science, Katholieke Universiteit Leuven; Celestijnenlaan 200a - bus 2402, B-3001 Heverlee, Belgium; 3 M3-BIORES, Department of Biosystems, Katholieke Universiteit Leuven; Kasteelpark Arenberg 30 - bus 2456, B-3001 Heverlee, Belgium

## Abstract

**Background:**

The intensive care unit (ICU) length of stay (LOS) of patients undergoing cardiac surgery may vary considerably, and is often difficult to predict within the first hours after admission. The early clinical evolution of a cardiac surgery patient might be predictive for his LOS. The purpose of the present study was to develop a predictive model for ICU discharge after non-emergency cardiac surgery, by analyzing the first 4 hours of data in the computerized medical record of these patients with Gaussian processes (GP), a machine learning technique.

**Methods:**

Non-interventional study. Predictive modeling, separate development (n = 461) and validation (n = 499) cohort. GP models were developed to predict the probability of ICU discharge the day after surgery (classification task), and to predict the day of ICU discharge as a discrete variable (regression task). GP predictions were compared with predictions by EuroSCORE, nurses and physicians. The classification task was evaluated using aROC for discrimination, and Brier Score, Brier Score Scaled, and Hosmer-Lemeshow test for calibration. The regression task was evaluated by comparing median actual and predicted discharge, loss penalty function (LPF) ((actual-predicted)/actual) and calculating root mean squared relative errors (RMSRE).

**Results:**

Median (P25-P75) ICU length of stay was 3 (2-5) days. For classification, the GP model showed an aROC of 0.758 which was significantly higher than the predictions by nurses, but not better than EuroSCORE and physicians. The GP had the best calibration, with a Brier Score of 0.179 and Hosmer-Lemeshow p-value of 0.382. For regression, GP had the highest proportion of patients with a correctly predicted day of discharge (40%), which was significantly better than the EuroSCORE (p < 0.001) and nurses (p = 0.044) but equivalent to physicians. GP had the lowest RMSRE (0.408) of all predictive models.

**Conclusions:**

A GP model that uses PDMS data of the first 4 hours after admission in the ICU of scheduled adult cardiac surgery patients was able to predict discharge from the ICU as a classification as well as a regression task. The GP model demonstrated a significantly better discriminative power than the EuroSCORE and the ICU nurses, and at least as good as predictions done by ICU physicians. The GP model was the only well calibrated model.

## Background

The intensive care unit (ICU) length of stay (LOS) of patients undergoing cardiac surgery may vary considerably. It is often difficult to predict, within the first few hours after admission, which patients will be discharged fast, and which will have a more prolonged ICU stay. This is problematic, not only for counseling patients or their relatives on the expected ICU LOS, but also with regards to bed and resource management in the ICU. Cardiac surgery risk stratification [1] models, as well as general ICU scoring systems [2] have shown to correlate with LOS. These models are based on pre- and postoperative risk factors, such as increasing age, impaired left ventricular function/ejection fraction, type of surgery, emergency vs. elective surgery, or the presence of pulmonary disease. Evidently, more complex surgery on a high risk patient has a higher probability for a prolonged LOS. The European System for Cardiac Operative Risk Evaluation (EuroSCORE) [3], Society of Thoracic Surgeons Score [4], the Parsonnet score [5], Cleveland Clinic Model [6], the Bayes model [7], and the Northern New England Score [8] are the most widely used systems [9]. EuroSCORE is considered to be the European "gold standard" regarding benchmarking, and has been shown to be predictive for LOS as a dichotomous variable [10-15].

Computerization in the ICU is on the rise [16]. A Patient Data Management System (PDMS) is a computerized ICU medical record that includes automatic registration of data from monitors and therapeutic devices, clinical observations, laboratory parameters, and drug therapy [17]. This large amount of data, including time series of some of these signals, is stored in a large relational database. Machine learning techniques can be used to analyze such a database in an automatic way [18]. In retrospective database analysis in a surgical ICU population, they have been shown to predict ICU LOS [19].

The goal of this study was to develop and validate a model that predicts ICU discharge of non-emergency adult cardiac surgery patients, by analyzing PDMS data from these patients. Predictions were made as early as possible after admission, by using data known upon admission of the patient, and trend analysis of vital parameters during 4 hours (which is the shortest interval that allows dynamic time series analysis). Machine learning methods were used to develop predictive models for a classification task, to predict the probability of ICU discharge the day after surgery (further referred to as 'second day of discharge prediction'), and for a regression task, to predict the day of ICU dischargeas a discrete variable (further referred to as 'day of discharge prediction'). Models were developed in one patient cohort; the predictive performance was evaluated in a separate and previously unseen validation cohort. The performance of the models was compared against the performance of the EuroSCORE, and against predictions done by the ICU nurses and physicians in the validation cohort, in order to have an indication whether in the future they could have a possible added value as clinical decision support tools.

## Methods

### Setting

At the Leuven university hospitals, all cardiac surgery patients are admitted postoperatively to the 56 beds surgical ICU, of which 22 beds are dedicated to the care of these patients. Decisions regarding patient discharge are made by the senior ICU medical staff. Once the decision is made that a patient is or will be ready for discharge, a bed on the cardiac surgery ward is always made available on the same day. For reasons of nursing staff on the regular cardiac surgery ward, discharge takes place between 11.00 AM and 19.00 PM. Therefore LOS, or day of discharge, is a discrete and not a continuous variable, and the minimum LOS is 1 day (admission day). The minimum criteria for ICU discharge after cardiac surgery are summarized in table 1.

**Table 1:** Guidelines for ICU discharge after cardiac surgery at the university hospitals Leuven

1. Respiratory criteria	The patient is extubated and weaned from mechanical ventilation or other forms of mechanical respiratory support. His oxygenation is good, or at least comparable to his pre-operative situation. To accomplish this, 3-4 liters of nasal oxygen supplementation is allowed. The patient has an adequate cough reflex and is able to maintain a safe upper airway.
2. Hemodynamic criteria	Inotropic support and vasopressor therapy have to be stopped upon discharge. No major arrhythmias compromising hemodynamic stability should be present. Atrial fibrillation is no contra-indication for discharge, provided an adequate rate control. Patients, who are depending on external epicardial pacing after surgery, where no background rhythm or implanted pacemaker is present, cannot be discharged to the nursing ward.
3. Neurologic criteria	Patient is awake, capable of communication and has sufficient pain control with his current analgesic therapy regimen.
4. Bleeding	No major bleeding, defined as a persistent need of transfusion of more than 2 units of packed cells per day.
5. Other organ systems	No vital threats to other organ systems (such as the kidneys, the central nervous system,...) are present

### Patients

The electronic medical records of scheduled adult (> 18 years) cardiac surgery patients, admitted to the ICU, were used for predictive modeling. Surgery was defined as 'scheduled' when the patient was called from home to undergo surgery. Heart transplant patients, pulmonary thrombendarteriectomy patients, patients on a left or right ventricular assist device or on extracorporeal membrane oxygenation, were excluded. The goal of the study was to predict ICU discharge to a normal ward, but death in the ICU was also regarded as discharge. The data of patients discharged to another ICU or to an intermediate care unit were not used.

The study protocol was approved by the hospital's ethical committee ("Commissie Medische Ethiekvan de Universitaire Ziekenhuizen KULeuven", Belgian reference number B3222006749), and the need for informed consent was waived.

In the medical literature, prospective validation of predictive models in a previously unseen dataset is the most generally accepted method [20]. Therefore, two cohorts of patients were selected: a development cohort of 461 consecutive, scheduled cardiac surgery patients, admitted to the ICU between the 18th of January and the 4th of December 2007, and a separate validation cohort of 499 consecutive patients, admitted between the 4^th ^of March 2008 and the 8^th ^of January 2009. Over the two time periods, there were no changes in surgical techniques, postoperative management, or in the cardiac surgical and ICU senior medical staff.

The baseline characteristics of validation and test cohorts are presented in table 2. Besides a significantly larger proportion of patients that underwent repeat cardiac surgery in the validation cohort (development: 27 patients (5.8%), validation 49 patients (9.8%), p = 0.023), there was no difference between the two cohorts.

**Table 2:** Baseline characteristics

	Test cohort (n = 462)	Validation cohort (n = 499)	p-value
ICU length of stay in days (continuous): median (P25-P75)	1.9 (1.2-3.6)	2.0 (1.2-3.9)	0.277
ICU length of stay in days (discrete): median (P25-P75)	3 (2-5)	3 (2-5)	0.360
Patients discharged on the day after surgery: n (%)	147 (31.8)	149 (29.9)	0.511
ICU mortality: n (%)	8 (1.7)	11 (2.2)	0.599
Hospital mortality: n (%)	15 (3.2)	16 (3.2)	0.972
EuroSCORE (additive): median (P25-P75)	5 (3-7)	5 (3-7)	0.202
EuroSCORE (logistic)%: median (P25-P75)	3.9 (2.1-7.4)	4.5 (2.2-8.1)	0.828
Type of surgery			
Isolated OPCAB: n (%)	190 (41.1)	179 (35.9)	0.094
Isolated on-pump CAB: n (%)	1 (0.2)	0 (0)	0.298
Valvular surgery (single, multiple and/or combined with CAB or other surgery): n (%)	250 (54.1)	298 (59.7)	0.079
Other cardiac surgery (congenital, aorta ascendens, myxoma ...): n (%)	21 (4.6)	22 (4.4)	0.919
Repeat cardiac surgery: n (%)	27 (5.8)	49 (9.8)	0.023
Surgery post-endocarditis: n (%)	7 (1.5)	7 (1.4)	0.885

### Database setup and input data

For the purpose of this study, a copy of the MetaVision^® ^(iMD-Soft^®^, Needham, MA, USA) PDMS database (Microsoft^® ^SQL) was made, and all data referring to the identity of the patients were removed from this copy. This included the patient's name, address, hospital identification number, and also all free text fields - because information potentially revealing the identity of the patient could be present in these fields. There were 5 parameter categories:

• *Admission data*: data that were known upon ICU admission, including the patient's history and pre-operative medical condition, the day of the week, and demographic data. Details regarding the type of surgery, when not available in MetaVision^®^, were taken from the Hospital Information System (HIS) or the ICU departmental database (FileMaker Pro^®^, Santa Clara, CA, USA). Missing numerical data were substituted by the population mean for that parameter. Missing categorical data were replaced by the value for that parameter that corresponded to a normal healthy condition.

• *Medication data*: type and cumulative dosage of drugs, intravenous fluids and blood products used during the first 4 hours in the ICU.

• *Laboratory data *of the first 4 hours in the ICU. The values of the laboratory parameters, and the 'count' as well as the 'trend' for repeated lab analyses were used. 'Count' is defined as the number of times a parameter is registered. Trend is a nominal variable, with three categories: when comparing consecutive values, does the value increase, decrease or remains the same within one standard deviation (SD).

• *Physiological data*: monitoring data (Merlin^®^, Philips^®^, Eindhoven, The Netherlands), mechanical ventilator data (Evita 2, 4, and XL, Dräger^®^, Lübeck, Germany) blood loss, and urine output, registered during the first 4 hours in the ICU. Monitoring and mechanical ventilation data are automatically stored in the database, at a resolution of 1 datapoint per minute. This way, 240 datapoints were present for these parameters. Blood loss and urine output were manually registered by the nurses, at least once every hour. Mean and variance of the monitoring data, and cumulative blood loss and urine output were used.

• *Dynamic data*: A 4 hours time series of 5 monitoring signals (systolic arterial blood pressure, pulse oxymeter arterial saturation (SpO_2_), heart rate, central blood temperature, and systolic pulmonary artery pressure) was analyzed. The length of this interval was chosen in order to have enough information on the dynamic evolution of the patients during their first hours in ICU. Artifacts were removed from the time series using a peak shaving algorithm, values exceeding 2 times the SD of the time series were removed. Missing values were linearly interpolated between two consecutive adjacent known values. Again, mean and variance were determined, but the time series was divided into 6 intervals of 40 minutes. This 40 minutes interval was chosen because by cross-validation in the development cohort, it provided the best predictive results, over a 30 or 60 minutes interval. Approximate Entropy (ApEn) was determined for the 4 hours time series of SpO_2_, systolic arterial blood pressure, and heart rate [21]. ApEn is a regularity statistic that quantifies the unpredictability of fluctuations in a time series. The 6 mean and 6 variance values for each of the 5 parameters, together with the 3 ApEn values, were used.

Overall, in the development cohort database 5.18% of data were missing; in the validation cohort the proportion of missing data was 5.74%. Variables with the largest amount of missing data were mainly related to pre-admission assessment of the patients, such as the pre-operative spirometry and pulmonary function tests (31-87% missing), pre-admission heart rate (35% missing), pre-admission blood pressure (27% missing). Intra-operative blood loss was not entered in the database in 50% of the patients.

### Predictive modeling

ICU discharge after scheduled cardiac surgery was predicted in two ways.

• Classification task: predict the probability of discharge on the day after surgery ('second day discharge prediction')

• Regression task: predict the day of discharge, which is a discrete variable (day 2, 3 ...) ('day of discharge prediction').

MatLab^® ^(The MathWorks, Natick, MA, USA) was used to build the predictive models.

Models were built for each parameter category separately, and integrated into a single predictive model. In a preparatory phase of the study, a logistic regression (LR) model and several ML models were developed for second day discharge prediction: decision trees (DT), Random Forest (RF), Support Vector Machines (SVM), and Gaussian Processes (GP). A review of the used methods can be found in [18]. The best model was selected by internal cross-validation in the development cohort.

The discriminatory power of the LR and DT models was unacceptably low, and GP proved to be the most performant of all models (results not shown). In addition to being highly performant, GP have a number of theoretical advantages such as the ability to deal with very complex datasets, and the possibility to identify the predictive variables (as opposed to black box models such as artificial neural networks) [22]. When making predictions, a model or mathematical function is applied to certain inputs to obtain an estimate of a certain output. In contrast to considering a single or a few optimal functions, GP give a prior probability to every possible function, with higher probabilities for the functions that are more likely, given the input data. In other words, GP are a distribution over functions and a natural generalization of a Gaussian Probability Distribution. This predicted distribution is obtained by taking into account non-linear interactions between the predictive variables to evaluate the similarity between the instances of the dataset. The obtained distribution assigns similar predictions to patients with similar values for their predictive variables, a behavior that reflects clinical intuition. GP were implemented according to the algorithms described by Rasmussen and Williams [23].

### Validation of the models

The developed models were tested in a previously unseen validation cohort. The performance of the *second day discharge prediction task *was evaluated by means of the area under the receiver operator characteristic curve (aROC) for discrimination. In advance, an aROC of above 0.70 was considered to be adequate [24]. The Brier score (BS) [25] (the average squared difference between the predicted probability and the true occurrence of a binary outcome), and the Brier score scaled (BSS) (a version of the BS that will give a more robust comparability of the accuracy of a model because it is not depending on population differences of the outcome), were also calculated. A Brier score should be as close to 0 as possible, with 0.25 as acceptable upper cut-off [26]. Reliability diagrams, where the predicted and observed relative frequencies are plotted against each other, were drawn [27]. These diagrams give a clear visual overview of the calibration or 'fit'. Finally, Hosmer-Lemeshow U-statistic was calculated as a measure of fit, with a p-value larger than 0.05 as an acceptable cut-off.

The performance of the *day of discharge prediction task *was assessed in several ways. First, predicted and actual median LOS were compared. Second, a loss penalty function (LPF), was calculated, which is the difference between the actual day of discharge (D_actual_) and the predicted day of discharge (D_predicted_), divided by the actual day of discharge.

$${\text{LPF=(D}}_{\text{actual}}-{\text{D}}_{\text{predicted}})/{\text{D}}_{\text{actual}}$$

This way, the relative error gets a less severe 'penalty' the further in the future this error is made. The LPF will take positive values if the predicted LOS is shorter than the actual LOS and negative values when the predicted LOS is longer than the actual LOS. Third, a prevalence plot was drawn, representing the actual and predicted numbers of patients discharged on each day. Fourth, root mean squared relative errors (RMSRE) were determined, with a value closer to 0 indicating a higher accuracy.

### Comparison with EuroSCORE

The additive EuroSCORE was calculated for every patient in the validation cohort using the research calculator available online [28]. The 17 EuroSCORE items were taken from the preoperative anesthesia record, available in the HIS. EuroSCORE was used for *second day discharge prediction*. For the *day of discharge prediction task*, we plotted EuroSCORE against LOS in the development cohort. The linear correlation equation obtained was then used to predict LOS in the validation cohort.

### Comparison with ICU clinicians

In the validation cohort, the ICU nurse and ICU physician caring for that patient were asked to perform the same two predictive tasks. Their predictions were collected prospectively and recorded in MetaVision^®^, by the Event Manager^®^. A pop-up window was programmed to appear 4 hours after admission to the first nurse and physician logging in to the patient's PDMS file, with the following questions. First, "What is the probability that this patient will leave the intensive care unit tomorrow, on the day after surgery?" The answer to this question is a probability between 0% (certainly not discharged on the day after admission) and 100% (certainly discharged on the day after admission). Second, "According to you, on what day will this patient be able to leave the ICU?" The answer to this question is a date. In order not to interfere with the clinical work, the pop-up could be overruled, but was repeated every 15 minutes. In advance, we determined that when the predictions were made later than 6 hours after admission, these results would not be used to compare with the GP models, as this would give the clinicians an unacceptable advantage of more than 2 hours of observation over the computer.

### Statistical analyses

Comparison of the baseline characteristics between the development and validation cohort was performed using Stat View^® ^5.0.1 for Windows (SAS Institute, Cary, NC, USA). A Chi-square test was used to compare between nominal variables, a Student t-test was used for the normally distributed, and the Mann-Whitney-U test for non-normally distributed continuous variables. Two sided p-values of less than 0.05 were considered significant.

For the *second day discharge prediction task*, aROC's and Brier Scores were calculated, and reliability diagrams were drawn. In order to compare between the aROC's, the DeLong method was used [29]. The Brier Scores were compared using a bootstrapping technique [26,30]. For the regression task, the Mann-Whitney-U test was used to compare LOS and LPF. Matlab^® ^was used as statistical software for the comparisons between the different models.

## Results

The GP models are accessible at http://www.kuleuven.be/licm/ml/gpdischarge1. The tables contain the input variables of the two predictive tasks and the coefficients, learned by GP, in order of their learned relative importance within each category.

### Second day discharge prediction

The validation results are summarized in table 3.

**Table 3:** Classification task

	GP (4 h)	EuroSCORE (4 h)	EuroSCORE vs. GP	Nurses (6 h)	Nurses vs. GP	ICU physicians (6 h)	ICU physicians vs. GP
**Validation cohort, n = 499**							
aROC	0.758	0.726	p = 0.286	X	X	X	X
Brier Score	0.179	0.324	p < 0.001	X	X	X	X
Brier Score Scaled	11%	0%	p < 0.001	X	X	X	X
Hosmer Lemeshow p-value	0.382	< 0.001	X	X	X	X	X
**Nurses answer ≤ 6 h, n = 396**							
aROC	0.769	0.726	p = 0.124	0.695	p = 0.018	X	X
Brier Score	0.177	0.326	p < 0.001	0.245	p < 0.001	X	X
Brier Score Scaled	13%	0%	p < 0.001	1.35%	p < 0.001	X	X
Hosmer Lemeshow p-value	0.405	< 0.001	X	< 0.001	X	X	X
**Physicians answer ≤ 6 h, n = 159**							
aROC	0.777	0.726	p = 0.334	X	X	0.758	p = 0.719
Brier Score	0.166	0.328	p < 0.001	X	X	0.216	p = 0.055
Brier Score Scaled	14.2%	0%	p < 0.001	X	X	12.5%	p < 0.001
Hosmer Lemeshow p-value	0.696	< 0.001	X	X	X	< 0.001	X

The interpretation of the different validation measures can be found in the methodology section.

In the validation set of 499 patients, the aROC values of the GP submodels built on the different data categories were 0.730 for the admission data, 0.690 for the medication data, 0.640 for the laboratory data, 0.710 for the physiological data and 0.670 for the dynamic data. The integrated GP model had an aROC of 0.758, a Brier Score of 0.179 and a Brier Score Scaled of 11%. The EuroSCORE had an aROC of 0.726, (p = 0.286 as compared to GP) but a Brier score of 0.324 and BSS of 0%. The GP model showed good calibration with a Hosmer-Lemeshow goodness of fit statistic (U-statistic) p-value of 0.382; EuroSCORE had a poor calibration, Hosmer-Lemeshow goodness of fit statistic (U-statistic) p-value was very low (p-value < 0.001).

In 396 of the 499 patients in the validation cohort, the nurses predicted discharge within 6 hours after admission. In this subgroup, the nurses' predictions revealed an aROC of 0.695 (p = 0.018 as compared to GP) and a Brier Score of 0.245. The aROC of the EuroSCORE model was not significantly different from the nurses' predictions (p = 0.379).

Only in 159 of the 499 patients a prediction by the ICU physicians was obtained within 6 hours after admission. In this small subgroup, the aROC for the physician's predictions was 0.758 (p = 0.719 as compared to GP, and p = 0.593 as compared with EuroSCORE), with a Brier Score of 0.216.

The Brier scores of the GP predictions were significantly lower (better) (p < 0.001) than those of the other predictions, except as compared with the physicians (p = 0.055). U-statistic was adequate for the GP models (p = 0.405), the EuroSCORE's, nurses' and physicians' predictions were poorly calibrated (p < 0.001).

**Figure 1:**
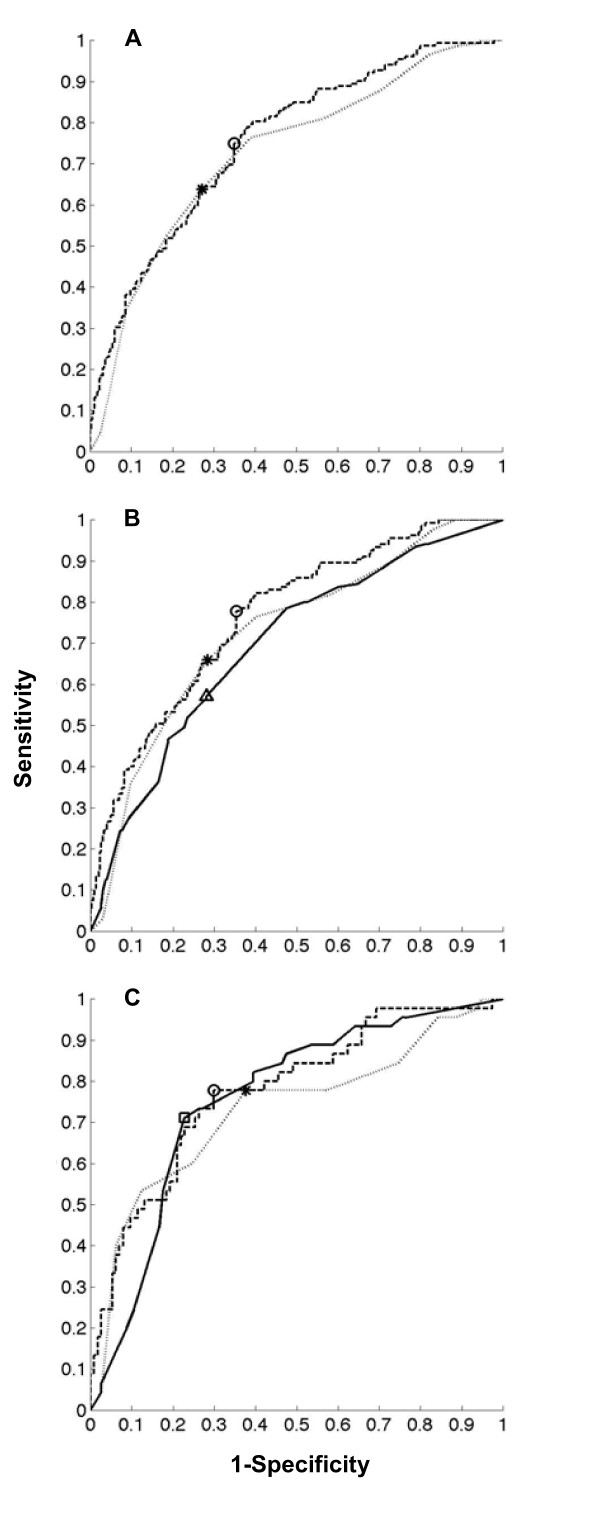
**ROC of the classification task**. *Panel A*. Validation cohort of 499 patients. ROC of the GP model (aROC = 0.758) (dark interrupted line), with a circle indicating the cutoff for best discrimination and calibration. ROC of the EuroSCORE (aROC = 0.726) (grey dotted line), with a star indicating the cutoff for best discrimination and calibration. There was no statistically significant difference between GP and EuroSCORE (p = 0.286). *Panel B*. Validation subcohort of 396 patients. ROC of the GP model (aROC = 0.769) (dark interrupted line), with a circle indicating the cutoff for best discrimination and calibration. ROC of the EuroSCORE (aROC = 0.726) (grey dotted line), with a star indicating the cutoff for best discrimination and calibration. ROC of the predictions by nurses (aROC = 0.695) (black uninterrupted line), with a triangle indicating the cutoff for best discrimination and calibration. The aROC of the predictions by nurses was significantly lower than the GP model (p = 0.018). *Panel C*. Validation subcohort of 159 patients. ROC of the GP model (aROC = 0.777) (dark interrupted line), with a circle indicating the cutoff for best discrimination and calibration. ROC of the EuroSCORE (aROC = 0.726) (grey dotted line), with a star indicating the cutoff for best discrimination and calibration. ROC of the predictions by ICU physicians (aROC = 0.758) (black uninterrupted line), with a triangle indicating the cutoff for best discrimination and calibration. The aROC of the predictions by ICU physicians was not significantly different from the GP model (p = 0.719).

**Figure 2:**
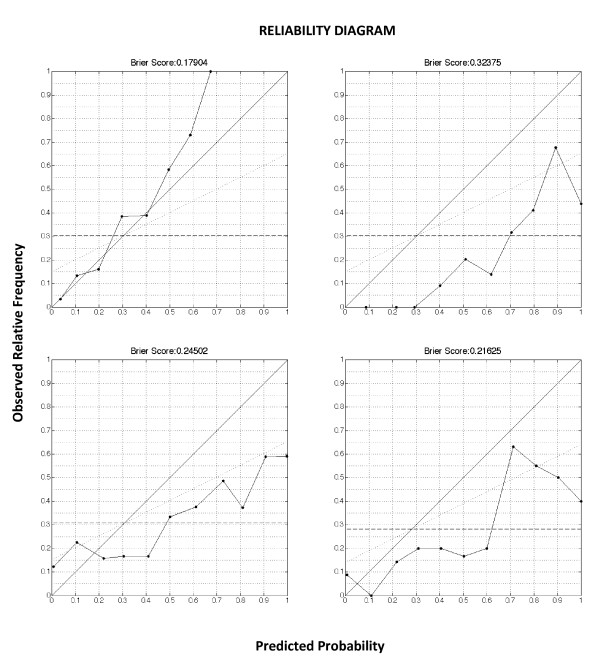
**Reliability diagrams of the classification task**. The reliability curve plots the observed fraction of positives against the predicted fraction of positives. The diagonal indicates a perfect reliability. The dotted horizontal line is the no resolution line, indicating the mean prevalence of the outcome in the population. The Brier score can be expressed as the sum of three terms related to the components of a reliability diagram. BS=reliability-resolution+uncertainty $BS=\frac{1}{N}\overset{K}{\underset{K=1}{\sum }}n_{k}{\left(p_{k}-o_{k}\right)}^{2}-\frac{1}{N}\overset{K}{\underset{K=1}{\sum }}n_{k}{\left(o_{k}-s\right)}^{2}+s{\left(1-s\right)}^{2}$ The first term, reliability, is the mean squared difference of the reliability curve to the diagonal. The second term, resolution, is the mean squared difference of the reliability curve to the no resolution line. The third term is a measure of uncertainty. *N *is the number of instances, *s *is the fraction of positives in the dataset, and for the *k*th bin, *n_k _*is the number of examples, *p_k _*is the predicted probability, and *o_k _*is the fraction of positives. *Upper right panel*. Validation cohort, 499 patients. Brier score and reliability diagram of the GP model. *Upper left panel*. Validation cohort, 499 patients. Brier score and reliability diagram of the EuroSCORE. Brier score was above the threshold of 0.25, and significantly higher (worse) than the GP models (p < 0.001). *Lower right panel*. Validation subcohort, 396 patients. Brier score and reliability diagram of the predictions by ICU nurses. Brier score was significantly higher (worse) than the GP models (p < 0.001). *Lower left panel*. Validation subcohort, 159 patients. Brier score and reliability diagram of the predictions by ICU doctors. Brier score was not significantly higher (worse) than the GP models (p = 0.055).

The aROC's are depicted in Figure 1. Figure 2 shows the reliability diagrams.

### Day of discharge prediction

The results are summarized in table 4.

**Table 4:** Regression task

	Actual	GP (4 h)	EuroSCORE (4 h)	EuroSCORE vs. GP	Nurses (6 h)	Nurses vs. GP	ICU physicians (6 h)	ICU physicians vs. GP
**Validation cohort, n = 499**								
LOS: Median (P25-P75)(days)	3 (2-5)	3 (2-3)*	4 (3-5)*	p < 0.001	X	X	X	X
LPF: Median (P25-P75)	X	0 (0-0.4)	-0.3 (-0.5-0)	p = 0.003	X	X	X	X
Patients with LPF = 0: n(%)	X	200 (40)	94 (19)	P < 0.001	X	X	X	X
RMSRE	X	0.408	0.643		X	X	X	X
**Nurses answer ≤ 6 h, n = 396**								
LOS: Median (P25-P75)(days)	3 (2-4)	3 (2-3)*	4 (3-5)*	p < 0.001	3 (2-3)*	p = 0.012	X	X
LPF: Median (P25-P75)	X	0 (0-0.4)	-0.3 (-0.5-0)	p = 0.002	0 (0-0.3)	p = 0.567	X	X
Patients with LPF = 0: n(%)	X	181 (46)	86 (22)	P < 0.001	152 (38)	P = 0.044	X	X
RMSRE	X	0.389	0.635		0.522		X	X
**Physicians answer ≤ 6 h, n = 159**								
LOS: Median (P25-P75)(days)	3 (2-5)	3 (2-3)*	4 (4-5)*	p < 0.001	X	X	3 (2-3)*	p = 0.578
LPF: Median (P25-P75)	X	0.2 (0-0.5)	-0.25 (-0.5-0.2)	p < 0.001	X	X	0.2 (0-0.4)	p = 0.755
Patients with LPF = 0: n(%)	X	59 (37)	27 (17)	p < 0.001	X	X	49 (31)	P = 0.234
RMSRE	X	0.439	0.631		X	X	0.612	

*p-value < 0.001 (as compared to actual LOS)

LOS= Length of stay

LPF= Loss Penalty Function

RMSRE= Root Mean Squared Relative Error

The interpretation of the different validation measures can be found in the methodology section.

**Figure 3:**
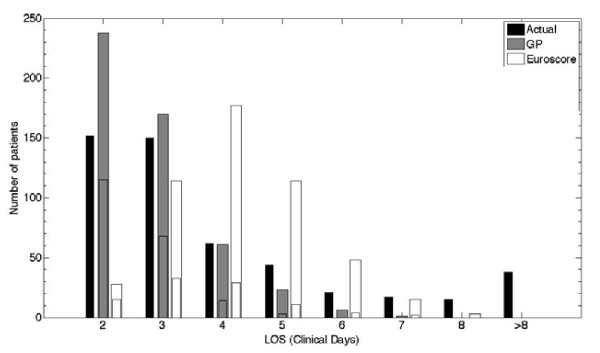
**Distribution of the predictions of the regression task (validation cohort, 499 patients**. GP versus EuroSCORE). The black bar indicates the actual number of patients discharged on each discrete day. Day 1 is the day of surgery (no patients were discharged on day 1), day 2 is the day after surgery, and so on. The other bars indicate the number of patients predicted to be discharged on that day. The subdivision in these bars indicates the number of true positives (predicted and actually discharged on that day).

In the validation cohort of 499 patients, the actual median (P25-P75) LOS was 3 (2-5) days. Median (P25-P75) LOS predicted by the GP models was 3 (2-3) days (p < 0.001), median (P25-P75) predicted LOS by the EuroSCORE was 4 (3-5) days (p < 0.001). The GP predicted day of discharge was significantly different from that predicted by the EuroSCORE (p < 0.001). The median (P25-P75) LPF of the GP model was 0 (0-0.4), in 40% of the patients the day of discharge was predicted correctly as indicated by an LPF of 0. The EuroSCORE had a significantly worse median (P25-P75) LPF ((-0.3 (-0.5-0), p < 0.001), and a significantly lower proportion of patients with LPF = 0 (19%, p < 0.001). Figure 3 is a visualization of the distributions of the predictions by the different models, as compared with the true day of discharge.

The nurses predicted a median (P25-P75) LOS of 3 (2-3) days in the 396 patients where they predicted within 6 hours, which was significantly different from the actual median (P25-P75) LOS in this cohort (3 (2-4) days) (p < 0.001). There was a significant difference between the predictions by the nurses and the predictions by GP (3 (2-3) days, p = 0.012) and EuroSCORE (4 (3-5) days, p < 0.001).

The median LPF of the prediction of the nurses and the GP model was not significantly different (p = 0.567), but the proportion of patients with an LPF = 0 was significantly lower (46% for the GP model, 38% for the nurses' predictions, p = 0.044).

In the 159 patients where the physicians made a prediction within 6 hours after admission, the actual median LOS was 3 (2-5) days, the physicians predicted a median LOS of 3 (2-3) days (p < 0.001), GP predicted 3 (2-3) days (p < 0.001), and EuroSCORE predicted 4 (4-5) days (p < 0.001). There was no significant difference between the predicted LOS by physicians and GP (p = 0.578), but EuroSCORE LOS prediction was significantly different (p < 0.001). There was no significant difference in median LPF or in the proportion of patients with LPF = 0 between GP and physicians.

In comparison to all other predictions, the GP model had the lowest RMSRE: 0.408 for GP compared to 0.643 for EuroSCORE in the 499 patients validation cohort; 0.389 for GP, 0.635 for EuroSCORE, and 0.522 for the nurses in the 396 patients cohort, and 0.439 for GP, 0.631 for EuroSCORE and 0.612 for physicians in the 159 patients cohort.

## Discussion

A GP model that used patient data of the first 4 hours after ICU admission, stored in a PDMS, was able to make accurate predictions on the probability of ICU discharge on the day after surgery, and was able to predict the day of discharge. The GP models were constructed in a development cohort, and tested in a previously unseen validation cohort. The GP model showed a significantly better discrimination than EuroSCORE and the ICU nurses, and was at least as discriminative as the ICU physicians. GP models were the best calibrated models, whereas the EuroSCORE, ICU nurses and physician's predictions showed overfitting.

In this era of computerized medical files, a large amount of patient information in the ICU has become available in an assessable format. Until now, only a few applications fully exploited the data-rich environment of intensive care, with its several information sources. Analyzing such large quantity of information, and using it for research purposes, remains a major challenge [18]. Since ICU clinicians use the data for vital decision making every day, it can be assumed that PDMS databases are important sources of information on the condition of ICU patients. A clinical PDMS database contains validated laboratory data, as well as clinical observations which are observer dependent, validated and unvalidated raw monitoring data, with artifacts and missing values. Several methods exist for inputting missing data; each of them has specific drawbacks. Replacing missing numerical data by the population mean for that value, as was done in the present study, might lead to regression towards the mean. Nevertheless, when a different imputation method was used, such as replacing missings by values corresponding to a normal healthy condition, the results did not change significantly (data not shown). For categorical data, the value that corresponds to a normal healthy condition was used to replace a missing value, which might have introduced some additional bias. Time series analysis in the present study was done after applying several low pass filters on the signal that have removed all high frequency components. We have done this under the assumption that the trend of a time series is more predictive for outcome than high frequency variability, very similar to the way doctors look at continuous parameters.

Although not set up to perform predictions, our results show that a GP model derived from these data was able to predict ICU discharge. At the population level, calibration and accuracy of the second day discharge predictions was good, with aROC well above and Brier score well below the predefined thresholds. At the individual level, the GP models showed to be the only well calibrated models. This is extremely important in clinical practice, when using the models for patient counseling. The exact day of discharge could be predicted in 40% of the patients, and the GP model showed the lowest RMSRE. Figure 3 shows that the GP tended to overestimate the number of patients discharged on the day after surgery, and underestimated the LOS in the longer staying patients. The relatively lower number of longer staying patients in the development cohort, upon which the GP models were learned, explains the higher uncertainty when predicting discharge in these patients.

This is the first study describing the use of GP for the prediction of length of stay in the field of medicine and intensive care. Currently, outcome prediction in the cardiac ICU is based on scoring systems that were developed and validated in large multicentre patient databases, such as EuroSCORE for a cardiac surgery patients [3], or APACHE for ICU patients [31]. These risk scores have been designed as benchmarking tools in order to compare different patient groups rather than for individual outcome prediction. APACHE IV uses data of the first 24 hours in ICU, and in cardiovascular surgery patients, there was no significant difference between mean predicted and observed ICU LOS, but aROC or calibration were not reported in this subgroup [32]. When using the additive EuroSCORE for the prediction of prolonged LOS, aROC's were 0.76, 0.72 and 0.67 when predicting a LOS of > 7 days, > 5 days and > 2 days respectively [14]. In the present study, the locally developed GP model outperformed the European gold standard risk stratification model, the EuroSCORE. In the classification task, this was demonstrated by the Brier score, which was unacceptably high in the case of EuroSCORE. In the regression task, EuroSCORE predicted a too long median LOS. It is well known that the EuroSCORE tends to overestimate the operative risk, especially in high risk patients. EuroSCORE was developed more than two decades ago, and in the case of mortality prediction, several authors have already proposed a recalibration [33,34]. Because the GP model was built on the local ICU database, it lacks the generalizability of the severity of illness scoring systems. Nevertheless, any computerized ICU could use the same methodology to build customized predictive models. Furthermore, such models can be systematically recalibrated over time, by relearning the models on an updated development cohort with more recent patients.

The GP models predicted better than the ICU nurses in the classification and the regression task. Although there was a trend towards a better performance than the ICU physicians, this was not statistically significant. When comparing the GP model with ICU nurses and physicians, one should realize that the clinicians had a few major advantages over the computer model. First, we did not obtain predictions within 6 hours in all 499 validation patients. This way, we lost statistical power. On the other hand, not being able to predict within time might be regarded as an extra evaluation criterion, as opposed to the GP model which was able to generate a prediction in every patient. Second, the predictions by physicians and nurses might have been biased in a sense that they could have postponed their predictions in the more difficult to predict patients whereas the GP models have always delivered a prediction within the allotted time, regardless of the uncertainty. Third, nurses and ICU physicians had an advantage of up to 2 hours of data over the GP model. In practice, it turned out to be impossible to respond immediately to the first pop-up window 4 hours after admission. Fourth, nurses and physicians will always have more information at their disposal than is present in the computerized chart of the patient.

A locally derived risk prediction model will not replace or compete with the validated scoring systems with regards to generalizability and benchmarking. Nevertheless, these locally developed models, based on the analysis of the computerized patient chart with machine learning techniques, have the potential to support the clinician in the care for critically ill patients. First, they could be the basis of an early warning monitor, which alerts the clinician when a patient deviates from the expected path with regards to his outcome. Second, in the same sense, they could be of use for the clinician when counseling a patient or his relatives to offer a realistic estimate of the expected clinical course of a particular patient. Third, the ICU LOS of a patient following cardiac surgery has, besides its medical and clinical relevance, also importance on the management level. ICU bed capacity is in many hospitals the bottleneck when planning cardiac surgery. In order to make optimal use of the available capacity, and in order to avoid an excessive number of patients that will die while awaiting cardiac surgery, a locally derived predictive model could be the basis of an ICU capacity planner. GP can take full advantage of the different information sources available in ICU, both static and dynamic, including therapy and the response to therapy. Although the discriminative power of the GP models is not significantly higher than the physicians, they can be of added value because they will deliver their predictions in a more reliable and consistent way (not postponing the predictions in the most difficult cases as the physicians probably did in the present study). In theory, each ICU could build its own predictive models based on its own patient database, because this methodology is able to take into account the specific local situation, and can be adapted and recalibrated over time. The results from this study should first be confirmed in other centers, and preferably in databases from multiple centers.

## Conclusions

Gaussian processes, a machine learning technique, are able to fully exploit a PDMS database and use all available data to build a predictive model for ICU discharge after cardiac surgery. This locally learned predictive model performed better than an existing scoring system (EuroSCORE), and better than ICU doctors and nurses. Machine learning offers a general method to learn the most performant predictive model for a specific context, database, or ICU. The predictive models, the used parameters and the weighed coefficients will be different in a different setting.

## List of abbreviations

APACHE IV: Acute Physiology and Chronic Health Evaluation IV; ApEn: Approximate Entropy; aROC: area under the Receiver Operating characteristic Curve; BS: Brier Score; DT: Decision Tree; EuroSCORE: European System for Cardiac Operative Risk Evaluation; GP: Gaussian Processes; HIS: Hospital Information System; ICU: Intensive Care Unit; LOS: Length of stay; LPF: Loss Penalty Function; LR: Logistic Regression; PDMS: Patient Data Management System; SD: Standard Deviation; SpO_2_: Pulse oxymeter arterial Oxygen Saturation; RF: Random Forest; RMSRE: Root Mean Squared Relative Errors; SVM: Support Vector Machine.

## Competing interests

The authors declare that they have no competing interests.

## Authors' contributions

GM and FG conceived and designed the study, drafted the study protocol, performed the statistic analysis, and drafted the manuscript. FG programmed, supervised and analyzed the machine learning algorithms. JR, MB and GVDB participated in the design of the study and helped to draft the manuscript. GM, DC and WDB programmed and designed the PDMS database, programmed the pop-up windows, carried out the database queries and de-identified the data. GM and DC calculated the EuroSCORE. KVL, JMA and DB did the dynamic data analysis. All authors read and approved the final manuscript.

## Pre-publication history

The pre-publication history for this paper can be accessed here:

http://www.biomedcentral.com/1472-6947/11/64/prepub
